# Supplementary material for: Efficacy of Plant Sterol-Enriched Food for Primary Prevention and Treatment of Hypercholesterolemia: A Systematic Literature Review
Source: Foods. 2022 Mar 15;11(6):839. doi: 10.3390/foods11060839 (PMC8954273; doi:10.3390/foods11060839)
Supplement: Supplementary file 1 [file foods-11-00839-s001.zip › ROB2_crossover_beta_v1.pdf]

Intention-to-treat

Unique ID	Study ID	Experimental	Comparator	Outcome	Weight	D1	DS	D2	D3	D4	D5	Overall
1	Baumgartner 2013	PS	Placebo	NA	1	+	+	+	+	+	+	+
2	Padro 2015	PS	Omega-3	NA	1	+	+	+	+	+	+	+
3	San Mauro Martin :	PS	Placebo	NA	1	+	+	+	+	+	+	+

+ Low risk
! Some concerns
- High risk

D1 Randomisation process
DS Bias arising from period and carryover effects
D2 Deviations from the intended interventions
D3 Missing outcome data
D4 Measurement of the outcome
D5 Selection of the reported result
