# Supplementary material for: Efficacy of Plant Sterol-Enriched Food for Primary Prevention and Treatment of Hypercholesterolemia: A Systematic Literature Review
Source: Foods. 2022 Mar 15;11(6):839. doi: 10.3390/foods11060839 (PMC8954273; doi:10.3390/foods11060839)
Supplement: Supplementary file 1 [file foods-11-00839-s001.zip › ROB2_IRPG_beta_v8 - Foglio buono.pdf]

Intention-to-treat

Unique ID	Study ID	Experimental	Comparator	Outcome	Weight	D1	D2	D3	D4	D5	Overall
1	Buyuktuncer 2015	PS	Placebo	NA	1	+	+	+	+	+	+
2	Cheung 2017	PS	Placebo	NA	1	+	+	+	+	+	+
3	Dong 2016	PS	Placebo	NA	NA	+	+	+	+	+	+
4	Ferguson 2019	PS	Placebo	NA	NA	+	+	+	+	+	+
5	Gagliardi 2010	PS	Placebo	NA	1	-	+	+	!	+	-
6	Lestiani 2018	PS	Placebo	NA	1	+	+	+	+	+	+
7	Mijares 2010	PS	Placebo	NA	1	+	!	+	+	+	!
8	Ras 2015	PS	Placebo	NA	1	+	+	+	+	+	+
9	Ruuth 2020	PS	Placebo	NA	1	+	!	+	+	+	!
10	Soderholm 2010	PS	Placebo	NA	1	+	!	+	+	+	!

+ Low risk
! Some concerns
- High risk

D1 Randomisation process
D2 Deviations from the intended interventions
D3 Missing outcome data
D4 Measurement of the outcome
D5 Selection of the reported result
