# Supplementary figures and images for: Efficacy of Plant Sterol-Enriched Food for Primary Prevention and Treatment of Hypercholesterolemia: A Systematic Literature Review
Source: Foods. 2022 Mar 15;11(6):839. doi: 10.3390/foods11060839 (PMC8954273; doi:10.3390/foods11060839)

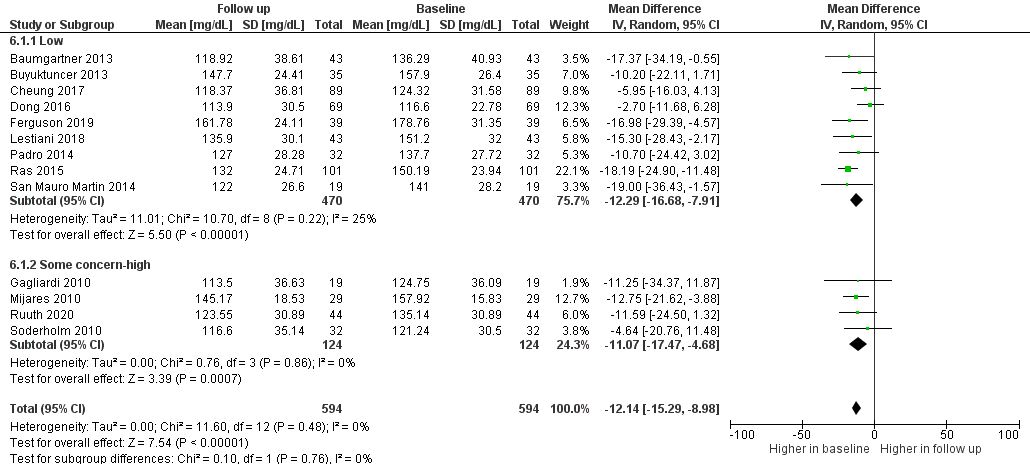

Supplement: Supplementary file 1 [file foods-11-00839-s001.zip › Forest plot.jpg]

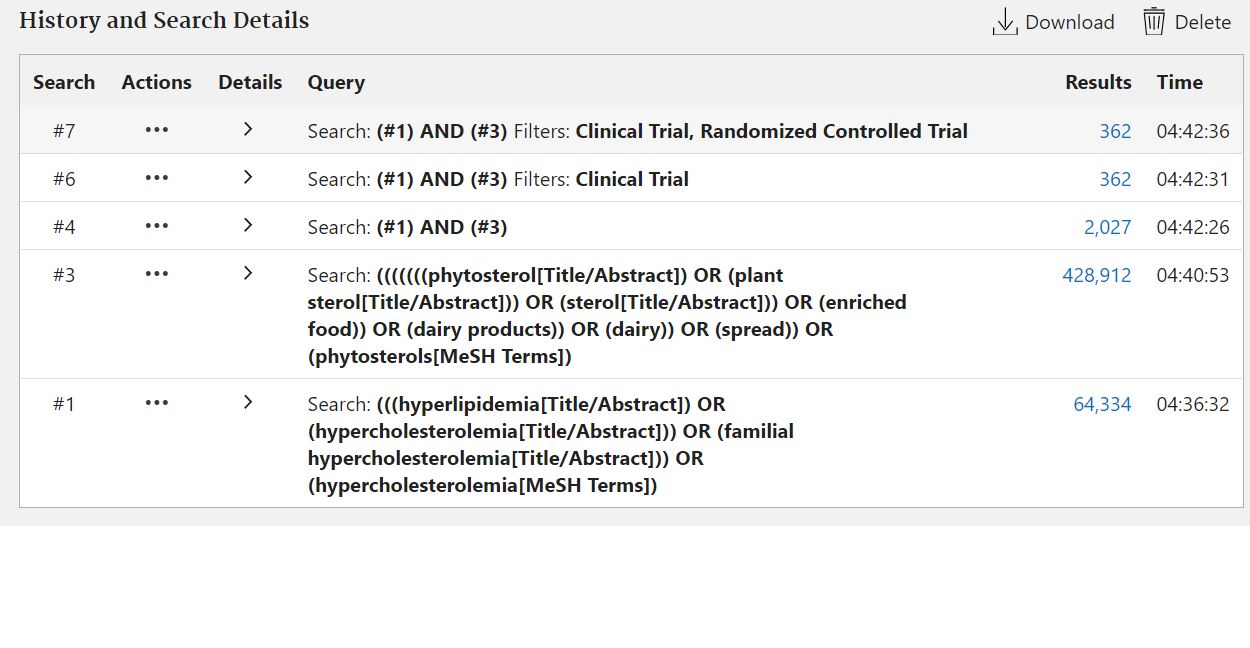

Supplement: Supplementary file 1 [file foods-11-00839-s001.zip › ricerca pubmed 22-04.jpg]
